# Illuminating humanist nature in teaching translation and interpreting studies: Devising an online customisable AI-driven subtitling course

**DOI:** 10.1057/s41599-022-01397-w

**Published:** 2022-10-17

**Authors:** Lisi Liang

**Affiliations:** Sun Yat-Sen University, Guangzhou, China

**Keywords:** Language and linguistics, Cultural and media studies

## Abstract

The paper sets out to devise an online subtitling course emphasising the human nature of translation. To gain a deeper understanding of audience behaviours and attitudes towards the consumption of subtitles, the article utilises questionnaires and case studies. Results are used to devise a learner-customised and technology-assisted online course about subtitling. The case studies that inspire the course content and design include the short-video platform Douyin (the Chinese equivalent of TikTok) and the comparison of two MOOCs, *Working with Translation: Theories and Practice* and *Consecutive Interpreting*. In summary, the course design for teaching subtitling will be based on the findings of the research questions and elastically adapted to the syllabus. We put forward our course with an emphasis on the practicality of producing subtitles in light of the technological acceleration in the marketplace and, more importantly, the humanistic nature of translation.

## Introduction

In recent years, unprecedented attention has been given to artificial intelligence (AI) technology and how it has reshaped teaching in translation and interpreting studies (TIS) (Díaz-Cintas and Szarkowska, 2020). Although research into the effectiveness of AI facilitation of teaching in TIS has still mainly been underdeveloped, Ye (2020, p. 3) argues that the utilisation of cross-disciplinary topics such as localisation, textual corpora, project management, and computer programming, etc., once seen as inherently not related to TIS, has now been reasonably integrated with TIS teaching. Nevertheless, the integration of these combined courses should not be implemented at the cost of the perspective on the essential human nature undergirding fields like TIS. Such a claim is in line with George Steiner’s argument that the human capacity for hermeneutics needs to be significantly enhanced instead of marginalised or even removed when employing technology-led methods (Steiner, 1992, pp. 240–246).

Given the significance of the essence of human nature within communication, the study of teaching subtitling has not been systematically explored. Interdisciplinary research is situated within the growing field of studies dedicated to the ever-expanding global phenomenon of teaching the use of translation technology. Under the ongoing and post-COVID-19 pandemic circumstances, it is imperative to anticipate a continued demand for online courses to avoid disruptions from lockdowns and to maintain quality teaching; for TIS, this includes filling in the gap of courses teaching AI-driven subtitling. Furthermore, an ever-expanding and fast-growing repository of audio–visual material requires strict selection criteria and most importantly, quality translations to cater to different target audiences.

Defining a customised online course on AI-driven subtitling is a prerequisite to meeting the requirement of a high-quality translation. It is an online course dedicated to learners who aspire to study and create bespoke subtitled audio–visual productions. Such subtitling practices are enabled by technologies such as machine translation, insertion of creative subtitles and postproduction, including revoicing and danmu/comments to promote the growth of cross-cultural intelligence and the sense of human community with a shared future.

## Research questions

To design a technologically advanced and learner-friendly online subtitling course, our course examines the advantages and disadvantages of these open online courses and short video platforms introduced later. Its overarching aim is to prepare students for the real-life subtitling professional practice that embraces technology-based and human-edited technology. Student subtitlers are able to work professionally in the current COVID-19 and post-COVID-19 environment. Focusing on human intelligence and technological advancement, our paper aims to improve student employability and future research in TIS with a particular emphasis on subtitling. To meet this end, two research questions are raised:

1. Why are the different case studies of short video platforms and online translation and interpreting courses comparable and suitable for our analysis?
2. How do these comparisons help devise an AI-driven and learner-led subtitling online course?

### Theoretical framework

Promoting a human-centred approach by devising an online subtitling course characterised by customisation and technological advances, this paper uses the polysystemic, multimodal and humanistic nature of translation studies as a conceptual framework. These concepts are suitable for analysing the case-study texts, which are abundant with polysystemic and multimodal semiotic resources, to better promote the core value of a human-centred focus in the tertiary educational setting. Polysystem theory is selected based on the heterogeneous and dynamic subsystems one has to consider when interpreting meanings, which matches the complexity of subtitling practice in the context of constantly evolving settings for a large number of audio–visual materials in the multimedia and 5G eras. Incorporating multimodality into a subtitling course enables significant use of novel content for a technology-facilitated subtitling course. These techniques include real-time danmaku, the user-oriented gift-giving action, and the replacement of written words/characters with emojis when leaving messages. This paper strives to emphasise the humanistic value of devising a subtitling course with the help of AI technology as both the act of communication and the human essence of interpretation in the translation process are mutually and equally stressed.

### Polysystem theory

The term *polysystem* was proposed by Itamar Even-Zohar (1978, p. 8) and can be defined as “a kind of mega-polysystem, the structure of which could explain relations hitherto neglected”. Díaz-Cintas applies polysystem theory to the study of Audiovisual translation (AVT), explaining it as “a group of semiotic systems that coexist dynamically within a particular cultural sphere” (Díaz-Cintas, 2004, pp. 22–23). In other words, AVT is not confined to linguistic elements of the target language. It is also shaped by the political, economic, social and cultural factors embedded in the language and culture of the target countries (Jin, 2018, p. 78; Liang, 2022). Furthermore, Díaz-Cintas (2004, p. 23) points out that a “new approach to translation allows for the translated work to be studied as a product in itself that is integrated into the target polysystem”. Built on the current application of the polysystem to AVT study, this paper intends to investigate how an AI-driven subtitling course heavily depends on the ecosystems in the Chinese tertiary educational and sociocultural context.

**Fig. 1:**
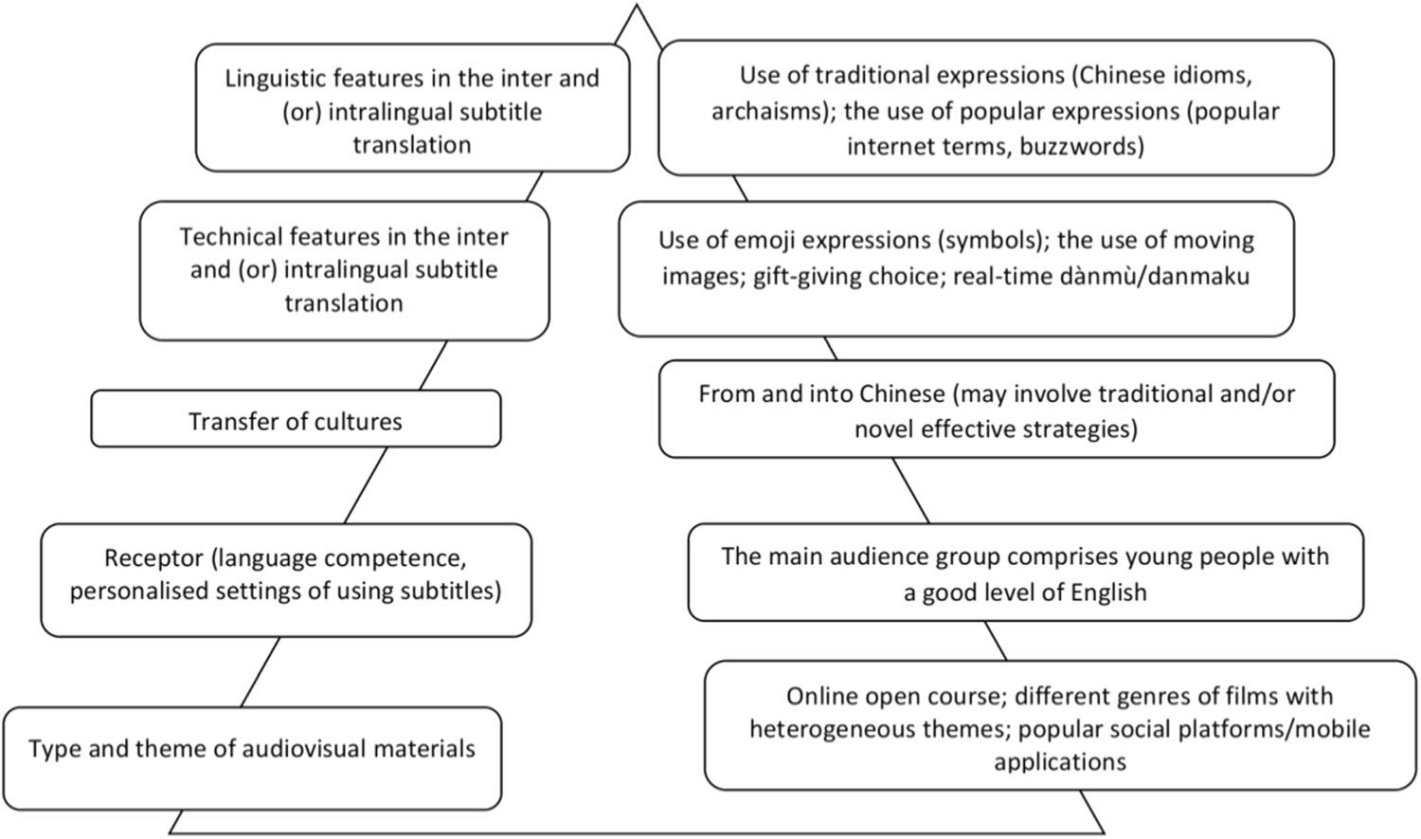
The connection between each factor and its counterparts in the polysystem in the case of designing an AI-driven subtitling course. This figure alters Jin’s (2018, p. 81) diagram pinpointing the connection between filmic factors and their counterparts under the guidance of polysystem to demonstrate the suitability and practicality of the present research explicitly in the context of devising a novel subtitling course.

Inspired by Jin’s (2018, p. 81) diagram pinpointing the connection between filmic factors and their counterparts under the guidance of polysystem, I alter her diagram to match the suitability and practicality of the present research explicitly in the context of devising a novel subtitling course (Fig. 1).

### Multimodality

If the above polysystemic structure provides a dynamic demonstration of subtitling practice as an internal system that is composed of language features, the transfer of technical preference, different cultures, receptors, and the genre and the theme of audiovisual materials to be translated, the application of multimodality targets the explicitly external elements of meaning-making. Multimodality is the combination of modalities in which many distinct semiotic resource systems interact in multiple and diverse ways in meaning-making (Baldry and Thibault, 2006, p. 21; Liang, 2020, p. 4).

This paper intends to break new ground in applying multimodality to designing a course on AI-driven subtitling by adding different user-led semiotic modes. It taps into the external environment of the course where technological and digital factors combine to make the course more appealing and effective to target learners. Audio and visual communication can be enhanced via visual and audio markers (Kress and van Leeuwen, 1996, p. 156).

### Humanistic nature in TIS

Apart from the audio and visual representation of multimodal markers used in interpreting meaning, this paper also applies Zinan Ye’s (2020) argument of preserving the hard core of human nature in the teaching of translation and interpreting studies. What he means by the “hard core” of humanities is the hermeneutic capacity of language users, or the ability to clarify uncertainty in language; it is “not pathologies of language but the roots of its genius” (Stenier, 1992, p. 246) that play “a centre of the fundamental element which cannot be removed or reduced” (Ye, 2020, p. 1). In line with this capacity, subtitling practice is seen as having the main responsibility to facilitate audience comprehension of the audio–visual material’s gist and plot (Ma, 2020, p. 9). However, the subtitler not only intends to render the meaning of the main plot but more importantly, to reproduce the aesthetic effect of the source. In other words, the process of subtitling is not simply word-for-word translation or “a passive reflection” of the source text (Matthiessen, 2001, p. 64) but an active art of recreation and reformulation to achieve equivalence of meaning with the original (Ma, 2020, p. 9). For Halliday (2010, p. 18), an effective translation is “the same function in the same context as the original”. Distiling human intelligence and care into the relevant and appropriate subtitling practice may bring pedagogical and sociocultural impacts to translation studies.

This paper attempts to bring humanities insights into devising a customised and AI-driven subtitling online course on two levels. On the one hand, the humanistic features in TIS characterised by our subtitling course relate to the situational level of different translation occasions. That is, the materials selected for pedagogical purposes should be thematically positive and humanistic in focus. On the other hand, the course draws on the broad sociocultural context that answers the call for developing a human community with a cognitively shared future. Chesterman’s (2002, p. 147) notion of translation causality may explain the rationale for these bilevel concerns because human beings can perform “this complex mental operation called translation”. Better translation means better effects on readers and intercultural relations in general (Chesterman, 2002, p. 148). The core of the humanistic nature of TIS in this online course on customised and AI-assisted translation is primarily concerned with establishing optimal causal conditions for both translations and training translators, with the ultimate goal of achieving better results, e.g., better effects on the shared future of human beings.

### AI-driven subtitling pedagogy

Translation is not a separate specialisation but a cross-curricular field (Damson, 2005, p. 111). Pym (1999, pp. 127–137) states that the inclusion of computer skills should be incorporated into the teaching of translation. To keep abreast of the latest developments in TIS with the cutting-edge technologies that cater to the new millennium, our course instils AI-driven technologies into the design of our virtual course with a particular focus on subtitling.

It is important to provide a definition of AI-driven subtitling in the design of our course. AI-driven subtitling denotes state-of-the-art technologies, including computer-assisted translation (CAT) tools and PC-associated skills, employed in devising the pedagogy for our online subtitling course. The CAT tool-enabled course uses specialised tools such as translation memory, automatic translation, and more general-purpose applications (Damson, 2005, p. 101). Specific PC-associated skills are required to meet the learning goals of the curricular design, including subtitle editing, online database systems, and webpage editing. (Damson, 2005, pp. 117–119).

The AI-driven technologies applied in our study adapt and innovate Wang’s (2017) three-stage translation technology applied through the whole translation process:

Before translation: format conversion, resource extraction, word count, repetition rate analysis, task analysis, term extraction, repeated segment extraction, and pretranslation.

During translation: auxiliary spelling and input, electronic dictionary references, parallel corpus query and verification, translation memory, term recognition.

After translation: quality assurance, format conversion, posttranslation typesetting, language quality assessment, and language asset management.

Based on the application of translation technology in three stages, the design of our subtitling course also undergoes three stages in terms of introducing cutting-edge technologies:

Before the subtitling course: terminology building, document formatting, genre-specific audiovisual text rewriting, choice-of-language selection, machine translating, presubtitling.

During the subtitling course: log-in face recognition, learning progress automatic tracking, users’ workstation configuration, creative emoji insertion, effectiveness feedback on the course through the creative economy, including gift-giving and rewarding, subtitle production and editing, using subtitling-led online platforms and software such as OOONA, Wincaps, Yicat, etc.

After the subtitling course: project management, postediting, postproduction, postsubtitling, localising terms, file converting, reviewing the project.

This has suggested that encouragement is needed to integrate AI-driven translation technology into the curricula (Alcina et al., 2007). Students majoring in TIS must be equipped with up-to-date serviceable skills to meet the expectations of the rising challenges in a digital environment (Damson, 2005, p. 101). The application of AI-driven technologies characterised by pre-during-post subtitling course design will be explained in the case analysis.

## Questionnaire

The classification of audiences is used to justify the necessity of detecting three tiers of audiences when reading subtitles (junior, intermediate, and senior audiences). The differences and similarities between the three tiers of audiences will be considered in the design of the subtitling course. The classification of audiences may allow translators to customise the viewing experience when devising innovative subtitles. This present study first employs a student-led questionnaire to justify the necessity and significance of adding personalised settings in the production of subtitles. Wray (2002, pp. 16–18) suggests that groups of words are stored in the mind as preassembled chunks to effectively understand written or spoken languages. In regard to subtitling, this implies that viewers can anticipate and recognise these prepackaged structures to process information/film plots more quickly (Pérez-González, 2015, p. 100). Subtitle settings, such as the completeness of interlingual, intralingual and/or bilingual subtitles (Liao et al., 2020), the size of the subtitles, the speed of audiovisual materials and the ending list of glossaries, are intended for viewers to choose from freely with the goal of optimising the viewing experience.

To test the validity of subtitling preferences, a questionnaire is conducted among college students. The subjects are 36 postgraduate students who majored in translation and interpreting in my multimedia translation module. They are in their first year of the programme Master of Translation and Interpreting (MTI) in the 2020 autumn semester at the School of International Studies at Sun Yat-Sen University. Their answers to the questionnaire are informed by their language competence and knowledge of the technology mentioned in the questions and are based on their preference for translation strategies (Jin, 2018, p. 16) and subtitling settings. Three questions and the corresponding multiple-choice answers are included in the questionnaire in Appendix 1.

**Fig. 2:**
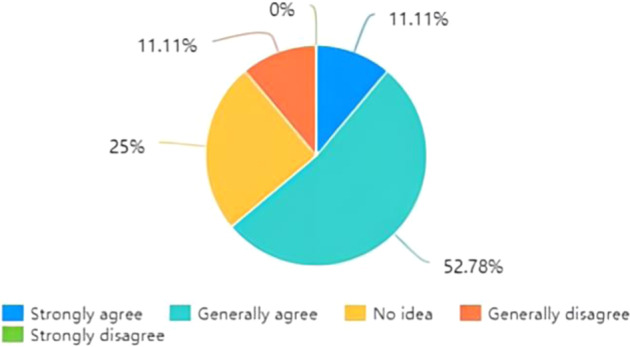
Preferences for settings for personalised subtitles. This figure underlines students’ preferences for settings for customisable subtitles in the context of the 2020 autumn semester’s multimedia translation course at the postgraduate level at the School of International Studies, Sun Yat-Sen University.

**Fig. 3:**
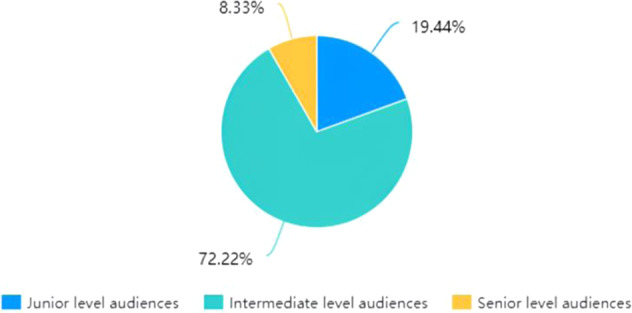
Self-classification of audience type. This figure demonstrates the self-generated taxonomy based on the assumption that the audiences referring to subtitled settings could be classified as junior, intermediate and senior audiences according to their language competence (in English and Chinese, in this context), and their familiarity with the topic.

**Fig. 4:**
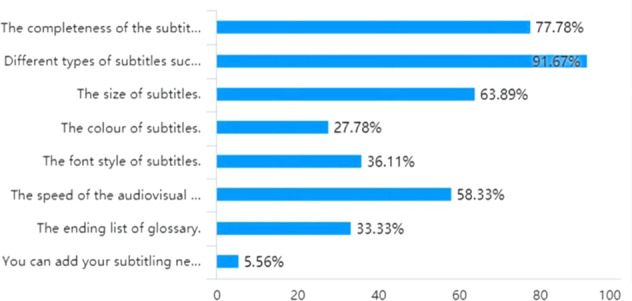
Selections of personalised subtitled options. This figure underlines the customisable subtitling settings to which the students aspire. For more details of the customised features that have not been shown fully due to the spatial constraints in the figure, please consult Appendix 1.

The focus of the questionnaire is on the necessity of devising more personalised settings for subtitling based on the self-classification of viewers, followed by ways in which the settings could be modified and added in a viewer-friendly manner. As Fig. 2 clearly illustrates, 63.89% of the participants agree that subtitling settings could be more personalised because most of them generally agreed with advancing a bespoke setting. Following this, nearly 75% of the postgraduate students in the multimedia translation course defined themselves as intermediate-level audiences who are deemed to have essentially mastered the foreign language and the filmic topic to a relatively satisfactory extent, while only 3 out of 36 participants regard themselves as proficient language learners at the senior level. Figure 3 shows that over 80% of graduate students pursuing a master’s degree in translation and interpreting in this class classify themselves at intermediate and senior levels. To meet audiences’ specific subtitle requirements, most students wanted to have different types of subtitles, such as bilingual, interlingual, and intralingual. One answer to the open question about preferred subtitling options reads, “If those videos on websites like Bilibili can offer subtitle options such as English/bilingual/none, subtitles would be much better.” This implies that graduate students are more likely to use popular social and streaming platforms such as Bilibili, Netflix, YouTube, and Amazon Prime (Díaz-Cintas and Aline, 2021, p. 16) and wish to have more freedom to choose the language and completeness of the subtitles. This also is reflected in the choice (Answers A and B to Question 3) of the two topmost attractive options in Fig. 4. Additionally, compared with the urge to adapt subtitle size and speed, other options, such as font style, an end list with glossaries and subtitle colour, are less necessary in the eyes of college students.

What Fig. 4 suggests is in line with Díaz-Cintas and Aline’s (2021, p. 16) argument that a more personalised and interactive set of features in subtitles can enhance the viewing experience. Moreover, these customised features can facilitate foreign language learning because they enable users to do the following:

lower the speed of the audio; adapt the subtitles to their language level by showing more subtitles in the viewers’ mother tongue if they are beginners and more in English if they are advanced speakers; or click on any word or expression to obtain its translation, definition and pronunciation (Díaz-Cintas and Aline, 2021, p. 16).

Expanding on Fig. 4’s result of adding glossaries of the relevant terminology, more options are advised specifically in terms of the definition of the key concepts involved and the pronunciation of certain words and expressions that are beneficial to language learners and watchers (Díaz-Cintas and Aline, 2021, p. 16).

Customised subtitle settings, on the one hand, can indeed facilitate language learning. On the other hand, it reshapes how users refer to them to explore more diverse and creative subtitling functions. Considering the technological advances pertinent to subtitling settings, Danan (2015, pp. 48–49) states that subtitled clips can be “manipulated in a variety of ways by users” due to the availability of a vast amount of audio–visual materials for download to personal computers. Such manipulation can be achieved by the settings on mobile devices used by the “multitasking, touch-screen generation”. Technological advances allow users to change their role from passive viewers to active content creators, helping them to choose among subtitling options such as adding transcripts and computer-generated subtitles so that these shared subtitles can be searched, downloaded and inserted into selected video clips (Danan, 2015, p. 51). This study encourages learners to use AI-driven technologies such as face recognition to memorise the kinds of users who watch and co-produce the subtitled work so that the effectiveness and efficiency of their subtitling work can be enhanced and customised.

Therefore, the audiences referring to subtitled settings could be classified as junior, intermediate and senior audiences according to their language competence (in English and Chinese, in this context), and their familiarity with the topic. In our case, this taxonomy is self-generated based on the student-led questionnaire. Such identification is vital because it can directly apply to language learning (Liao et al., 2020). It is also in line with our objective to optimise the viewing experience by offering the audience more freedom to select personalised subtitled settings.

## Case analysis

After confirming the necessity and importance of customised subtitles, we will analyse one popular social platform and two translation-led online courses for designing our subtitling course. This is because they cover the specificities and complexities in recreational and educational contexts featuring subtitling practices that manage a plurality of genres targeted explicitly at different text types (Díaz-Cintas and Aline, 2021, p. 61). As a result, these three multimodal texts are used as the corpus of this study. They include one of the most popular social platforms of its kind, Douyin, and two well-received translation-related classes from the West and China, *Consecutive Interpreting* and *Working with Translation and Interpreting*, respectively. As Matthiessen (2001, p. 64) put it, translation functions as “a creative act of reconstructing the meaning of the original”, and the process for devising the subtitling course takes such creativity as a starting point to combine different semiotic systems and resources suitable to domestic and international subtitling learners.

### Douyin

The two most successful versions of Douyin are used in this section to explore a more personalised setting for teaching subtitling. In total, three Douyin mobile applications are circulating in the market: Douyin, Douyin Jisu (fast-speed) edition and Douyin Huoshan (which has a volcano as its application logo) edition. The first two are more popular than the last one among users, with 37.6 billion and 10.7 billion mobile users, respectively (data accessed 05/09/2022). The data indicate that these Douyin versions are well-received among Chinese users. I will analyse the similarity and distinctions between these two successful editions of Douyin, focusing on specific personalised settings that could be applied to teaching subtitle creation. Both Douyin and Douyin Jisu (hereafter “Jisu”) editions are domestic short video platforms owned by the Chinese tech giant ByteDance (Kaye et al., 2020, p. 230). Unlike their international counterpart, Tik Tok, these three editions mainly target the Chinese market, with short video durations from 15 to 60 s (Kaye et al., 2020). With the shared goal of social entertainment (Su, 2019), these platforms have similar digital architecture and appearance but are marketed to users with different needs. Three main distinctions^1^ and advantages between these two Douyin editions are summarised below:

1. Technologically, compared to Douyin, Jisu requires less memory for its smaller installation package and less network bandwidth, so its operational speed is faster.
2. Economically, Jisu provides its registered users with more economic value when browsing short videos by sending out bonuses with real money that could be directly transferred to users’ Douyin bank accounts. Additionally, there are diverse ways for users to earn money, such as sending invitations to new members, watching livestream, counting daily steps, receiving a meal allowance, reading novels, and playing online games, etc.
3. From the aspect of the mobile application interface, the Jisu edition of Douyin is advertisement-free, which minimises disturbances to the viewer’s watching experience.

These three distinctions help both versions stand out technologically, economically and creatively, which is highly relevant to subtitling as inspiration for user-friendly settings. Localised settings, such as the speed of the audio, flexible language choice, completeness of subtitles according to viewers’ language competence, and the definition and pronunciation of certain words and expressions are all considered (Díaz-Cintas and Aline, 2021, p. 16); similarly, the course must advise on subtitle size, font, glossaries and colour to provide an optimal viewing experience. According to the different advantages of the two Douyin versions, options for a smaller installation package, less network bandwidth and faster operational speed could optimise the user experience. A subtitling course that engages with the creative economic value of these platforms could financially benefit student users if their subtitled work is easily uploaded to match with short videos. Then, it can be arranged that both viewers and subtitle makers receive bonus money when the work is viewed or liked. The creative act of producing new forms of subtitles with the economic value given by the platform is a key example of practising and participating in a creative economy. The notion of a creative economy can be understood as a creative objective (Douyin short video) attached to economic value (bonus money awarded to both video creators and viewers) (Howkins, 2002, p. 4). In this way, cross-disciplinary fields, including translation studies and creative economy, are used to understand subtitling practice in a broader sociocultural polysystemic structure.

Therefore, one of the aims of the subtitling module is to first provide an overview of the specific contemporary technology-oriented settings at play in creating subtitles. Second, the course must consider the intended user-friendly function of subtitling and the different potential needs of target audiences. Third, we reinforce the importance of sustainably creating and uploading positive and human-centred content in short videos online.

Taking these objectives into careful consideration, in the course, students are first able to identify key localised subtitle settings among new trends in subtitling by looking at representative cases from popular short video platforms in China and the West. For instance, Kuaishou, Douyin and Meipai are three major players tailored to Chinese target audiences (Su, 2019), while the Little Red Book targets younger Chinese females (Guan and Wen, 2021). From the West, popular short video platforms will be shown to students, e.g., Snapchat, Instagram and Musical.ly (Kaye et al., 2020, p. 230). Furthermore, user-friendly settings and technological advancements drawn from these Douyin-related applications bring insights into course design. These creative and technical parameters demand significant attention to “the myriad layers of meaning beyond words” (Jin, 2018, p. 77) when applied to audiovisual pedagogy. In the modern era, creative localisation and technological considerations are helpful to attract learners within the polysystemic target culture. Finally, it is of great importance to understand how thematically different short videos affect humanity physically and spiritually. The translation and the expected effect of the uploaded short video should be positive and promising. The rationale behind the above requirement is twofold. Individually, the beneficial impact of the social media consumed by netizens daily can bring long-lasting and profound improvements to individual welfare (Allcott et al., 2020, p. 631). Collectively, social media’s impact on welfare enables a trajectory towards “optimism about potential benefits” instead of “widespread concern about possible harms” (Allcott et al., 2020).

### Consecutive interpreting

Conventional subtitles are insufficient to fully cater to modern viewers according to the previous questionnaire. Student opinions were also collected through independent and group presentations from the Massive Open Online Courses (MOOC) Consecutive Interpreting^2^ to analyse the suitability of inventing personalised and AI-driven subtitle settings. One of the key features of Consecutive Interpreting is the frequent use of student demonstration and interactive feedback between students and teachers which make it different from other distance courses. To date, approximately 24,000 learners have participated in this online course (data accessed 28/05/2022) since its tri-annual sessions in 2018; it changed to yearly sessions in 2019. I took part in its 2019 session in which heavy loads of homework and practical student demonstrations left me with a deep impression. This 16-week course also included tasks after each weekly course and a final examination.

**Fig. 5:**
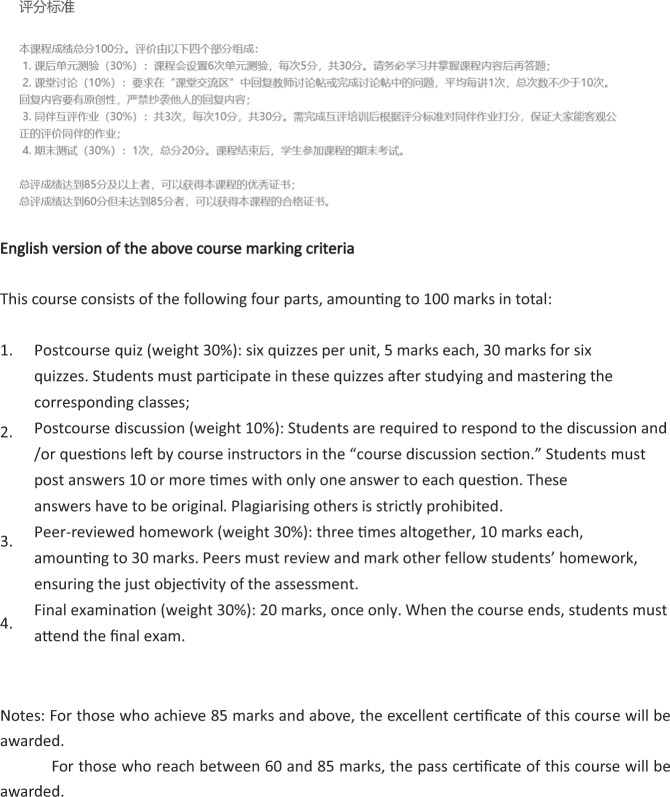
Marking criteria of the course in Chinese and its English translation. The detailed assessment in Chinese of the online course Consecutive Interpreting by Guangdong University of Foreign Studies and its English translation.

Figure 5 underlines the strict criteria in which marks can be obtained, but also their diversity ranging from multiple-choice quizzes (5 marks each, 6 times in total, weighted to 30% of the final grade), after-class discussions through comment submissions (1 mark each, 10 times in total, weighted to 10%), peer-reviewed marking (10 marks each, 3 times in total, weighted to 30%) and a final assessment (weighted to 30%). It is also stipulated that those awarded over 85 marks can receive a certificate with distinction. In comparison, those who receive 60–85 marks will only receive a certificate with a pass. Although the CI course homework is systematically classified by different questions and themes, our subtitling course will be organised around a less stressful method of learning to cater to domestic and international learners. However, the organisation of this mark-oriented module polysystem could be a reference when we design the mid-term and final assessments. One striking weakness of the CI course is that students are required to leave comments to obtain marks (10%), which may lead to negativity and pressure among learners who do not necessarily feel they have questions worthy of raising. Although there are areas for improvement in the CI course, it is advisable to follow its learner-based structure for each session. The learning objective of the session will be introduced at the start of class, followed by lectures and student demonstrations, and end with a summary of what has been taught.

The in-course student demonstration is an excellent example in which the student as a role model will make authentic mistakes as ordinary students do. Such authenticity may encourage average learners to feel more closely aligned with their classmates, particularly beginners. In this way, the student-led, real-time demonstration is fully incorporated into the self-study of distant learners, which is a good design for use with target learners (Thompson, 1996, pp. 206–207). The higher level of interaction and interpersonal commitment are retained even among distanced learners and teachers (Chang, 2018, p. 174), although features of face-to-face interaction in traditional teaching are lost.

**Fig. 6:**
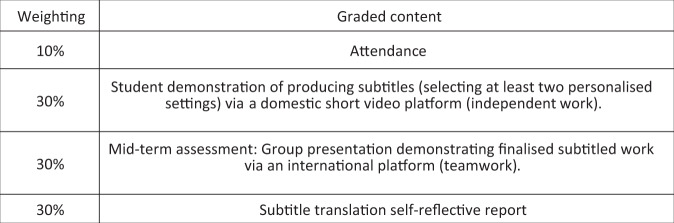
Assessment of the subtitling course. This figure indicates the assessment of the Online Customisable AI-driven Subtitling Course.

Hence, in constructing our subtitling course, except for the personalisation of subtitled settings, student demonstrations are pertinent for modelling how to produce AI-driven subtitles. Since learners can pick up from where they have left off from the previous session, the subtitling course can also memorise users’ preferences in audio–visual materials and prioritise their subtitled settings so that the viewing experience can be further enhanced. This enhancement could be achieved with the help of artificial intelligence technology by collecting and analysing user data. With facial recognition log-ins, access to the course will be made faster to save time, and it will be at the learner’s convenience to continue customising their subtitle settings and learning process while producing subtitles. Furthermore, building on the learners’ familiarity with popular video platforms at home and abroad, the student demonstrations can target each representative platform to design creative subtitles for use with each of these social platforms; ideally, this work will be attached to economic value if positively recognised. Last, adapted from the assessment of the CI course, student subtitling will be assessed in a much more relaxed manner to compensate for any perception that the workload is relatively heavy, which may distract the learner’s attention. Hopefully, our course will be equally fruitful by implementing the assessment, as Fig. 6 indicates.

In summary, the scientific organisation and assessment style for the subtitling course will be based on the favourably received Consecutive Interpreting MOOC. The key to the CI course is its high interactivity and connectivity with students that will be imitated and modified in our course. In addition, the oft-dreaded, intensive postcourse assignment will be replaced by open-ended and reflective written report. In light of this, the teaching polysystem is affected by “scheduling constraints” (Jin, 2018, p. 82) aiming at target learners who desire more desirable learning attainment with a relatively smaller physical workload and less mental stress. Such considerations built around the design of less-stressful assignments are in line with “humanistic therapies”, which deal with “person-to-person relationship with a particular focus on emotion and experience to address depression, anxiety, minor adjustment difficulties and relationship difficulties alike” (Elliott, 2002, pp. 58–75). The effectiveness of humanistic therapies applies to the design of courses in that they both inculcate a respect for “uniqueness and individuality” to achieve a positive functional and friendly environment (Elliott, 2002, p. 75). The use of self-reflective reports instead of intensive homework assignments ensure “feasibility and desirability” throughout the study process (Elliott, 2002, pp. 74–75).

### Working with translation and interpreting

Having understood the effectiveness of applying human-centred theories to teaching translation and interpreting, we use the next MOOC to facilitate the ability of target learners to link knowledge gained in previous sessions with personalised subtitling settings and the application of these settings in the making of actual AI-driven subtitles to thematically diverse topics in the subtitling course.

**Fig. 7:**
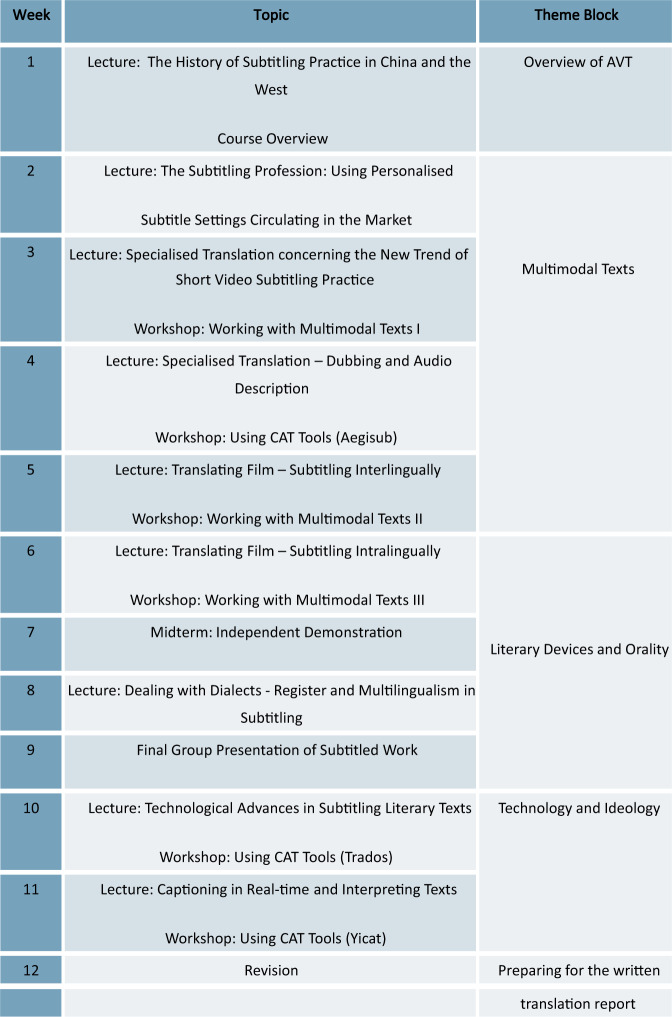
The schedule for the subtitling course. This figure demonstrates the relevant topics incorporated into the discussions of subtitling endeavours in our course.

As one of the pioneering online courses at the heart of translation studies on the online platform Futurelearn, produced by the School of Modern Languages, Cardiff University, *Working with Translation: Theories* and *Practice*^3^ was launched by the Open University, included among the elite UK and international universities^4^. With almost 55 thousand distant learners enroled, this course has been successfully rated 4.8 out of 5 by students. I once served as a tutor and mentor for this course after 2019. My primary duty was to provide feedback to subscribed learners who raised associated inquiries while enroled in this 4-week course. This provided valuable insights into the design of an online course tailored for contemporary learners by capturing the digital footprint of learners using the technology. This worldwide technology (WWT) course is characterised by instructional methods with video presentations, short quizzes and integrated gamification elements in the learning process over a wide range of translation-related topics. The diversity of the topics ranges from the definition of translation, types of translation, the translation profession, specialised translation, literary translation, and professional interpreting to, beyond the profession, bilinguals and multilingual translators. These relevant topics could be incorporated into the discussions of subtitling endeavours in our course, as Fig. 7 demonstrates.

Intriguingly, the after-course comments shown in the WWT course are highly interactive and thought-provoking. I quote some of them below to inspire the production of our subtitling course.

In response to the question “What is translation?” the most-liked comment was written by Alessia Mosca, who left this comment on 15 January 2019:

… That’s what translation is: moving across borders and overcoming barriers.

This is the reason why I love my job: it allows me to explore new worlds and understand them, having the possibility to explain them to others. It enriches me and helps me to enrich other people. Translation allows me to know and create.

Mosca’s comment provides greater detail on how translation can be a form of personal enrichment regardless of linguistic, geographical and cultural barriers. Translation entails communicating with other individuals, understanding a culture, transferring messages, and sharing past experiences and future desires, etc. However, some comments can broaden our horizons by offering contractionary and controversial views that are open to debate:

This was contributed by Rosalind Alcock on the same date under the same question:

*The translation is the act of accurately crafting one language into another to the effect that neither meaning nor style is lost and the reader feels a native speaker wrote it*.

The lead educator of this course, Professor Loredana Polezzi, replied and sparked follow-on discussions in identifying the authenticity and originality of the translation.

I like how you use ‘crafting’: it speaks of care, effort, ability…

I am not sure all native speakers are the best possible model for translation, though…:-(

Loredana even uses an emoji facial expression “:-(” at the end of her reply to express her doubt and uncertainty regarding the sweeping comment made by Rosalind that a translation can be reshaped to fit the imagery put forth by all native speakers. I agreed that leaving comments on questions worth discussing that are not mandatory can spark increased reflection. In this WWT course, there are fewer compulsory quizzes than in the CI course, which lessens learners’ burdens. Taking this into account, the AI-driven subtitling course will not use compulsory closed-book tests. Instead, the more creative and user-led independent and group subtitling work will be assigned for learners to explore in the actual subtitling world.

In summary, this instance is cumulatively supported by polysystem, multimodality and human nature. Polysystem is integrated into the subtitling course design in different subareas of translation, such as translation technology, specialised translation within multimodal texts, film translation, literary translation and interpreting studies. The combination of these will sufficiently enable discussion of the suitability of the planning of our course. In terms of the instructor use of the symbol of a dissatisfied face in an after-class discussion, the resources of symbolic facial expressions in the form of emojis interact vividly with different target learners across cultural borders (Kress and van Leeuwen, 1996, p. xvi). Last, the considerate call made by one of the students enroled in the MOOC Working with Translation and Interpreting to complete our life through the act of translation again reflects the humanistic value of maintaining a positive and promising outlook for “self-reflective investigation” (Elliott, 2002, p. 75).

## Conclusion

In response to the two research questions set at the beginning of this paper, first, the suitability and feasibility of drawing course design from one short-video platform and two online translation-led courses as key case studies heavily depends on their successful reception and massive popularity among viewers. Each of these texts features new forms of subtitles with technological advances and creative economic values suitable for application to the customised subtitling course. In addition, my experience and knowledge in the Working *with Translation: Theories and Practice* course as a researcher as well as a mentor can shed some light on the creation of the subtitling course. These texts are thereby selected as the corpus of this study.

Second, this overarching question is related to the significance of the study. It explores how a technology-enabled and user-friendly online course on subtitling cannot be devised at the expense of human creativity and shared well-being. Although technology can facilitate the effectiveness of course-related exercises and enhance learning attainment, selecting the thematically diverse content of the audio-visual materials is more important for relaying the human-centred philosophy of the pedagogical context. Specifically, in designing a learner-led course on AI-driven subtitling suitable for online learners, I identified course objectives first and presented them to students to help identify the trends of personalised subtitle settings. Then, the specific outline of this 12-week course is presented with thematically diverse and immediately applicable topics. The assessments for each module are flexible and presentable so that learners achieve course objectives by producing technologically advanced, up-to-date, localised and aesthetically accessible subtitles, which are attached with creative economic value that might be readily realised through uploads onto short-video platforms. Artificial intelligence technology will be combined with CAT tools such as Aegisub, Trados and Yicat to produce subtitles, and applied to broader platforms in which face recognition technology is used to memorise account settings and big data technology is used to collect user preferences and update audio–visual materials regularly.

Ultimately, the humanist nature of translation will be elastically instilled into the course when human intervention may be needed for translating themes or discourses relating to a greater community of human beings. The expected outcomes of this online course enable users to take advantage of artificial intelligence and their understanding of human nature for customising technology-enabled subtitles.

## Supplementary information

Appendix 1

## Data Availability

The datasets generated during and/or analysed during the current study are available from the corresponding author upon reasonable request.

## Footnotes

1 Websites illustrating the differences between these two short video platforms are shown as follows: (1) https://www.hackhome.com/info/504752.html, (2) https://www.cncnzz.com/news/19-1484.html, and (3) https://www.chinasongzhuang.cn/article/10422.html

2 This instance of *the Consecutive Interpreting* MOOC class is available on the web-based instructional site: http://www.icourse163.org/learn/GDUFS-1002493010?tid=1206122222#/learn/announce.

3 An example of an MOOCs-style Futurelearn classroom, *Working with Translation: Theories and Practice* is accessible online: https://www.futurelearn.com/courses/working-with-translation.

4 The new company, Futurelearn, released its first online courses in September 2013, attracting more than one million subscribers. It is among the MOOCs listed to provide distance learning for learners throughout the globe (MOOC List, 2012–2019).
