# Supplementary material for: Illuminating humanist nature in teaching translation and interpreting studies: Devising an online customisable AI-driven subtitling course
Source: Humanit Soc Sci Commun. 2022 Oct 17;9(1):378. doi: 10.1057/s41599-022-01397-w (PMC9575621; doi:10.1057/s41599-022-01397-w)
Supplement: Supplementary file 1 — Appendix 1 [file 41599_2022_1397_MOESM1_ESM.pdf]

Appendix 1 Three questionnaire questions

(1). To what extent do you agree that subtitling settings could be more personalised?

A. Strongly agree.
B. Generally agree.
C. No idea.
D. Generally disagree.
E. Strongly disagree.

(2). Suppose that audiences who resort to subtitles according to their understanding and knowledge of the filmic topic were classified into three tiers (junior, intermediate and senior-level audiences) when watching audio-visual materials. Junior-level audiences need subtitles the most and can barely trust their listening and comprehension skills; intermediate-level audiences have mastered the foreign language and the filmic topic to some extent while senior-level audiences can appreciate the audio-visual materials mostly without referring to the subtitles. How do you classify yourself into the above three tiers?

A. Junior-level audiences.
B. Intermediate-level audiences.
C. Senior-level audiences.

(3). Which of the following subtitling options do you think should be added to improve the functionality of subtitles to enhance the viewing experience?

A. The completeness of the subtitles can be chosen according to the different needs of audiences.
B. Different types of subtitles, such as bilingual subtitles, interlingual subtitles, and intralingual subtitles, can be chosen according to different needs of audiences.
C. The size of subtitles.
D. The colour of subtitles.
E. The font style of subtitles.
F. The speed of the audio-visual materials can be adjusted according to the different needs of audiences.
G. An ending glossary of terms.
H. You are welcome to add your preferred subtitling needs.
